# Supplementary material for: Comparative Analysis of Microbial Communities in Diseased and Healthy Sweet Cherry Trees (Prunus avium L.)
Source: Microorganisms. 2024 Sep 5;12(9):1837. doi: 10.3390/microorganisms12091837 (PMC11433754; doi:10.3390/microorganisms12091837)
Supplement: Supplementary file 1 [file microorganisms-12-01837-s001.zip › microorganisms-3185379-supplementary.pdf]

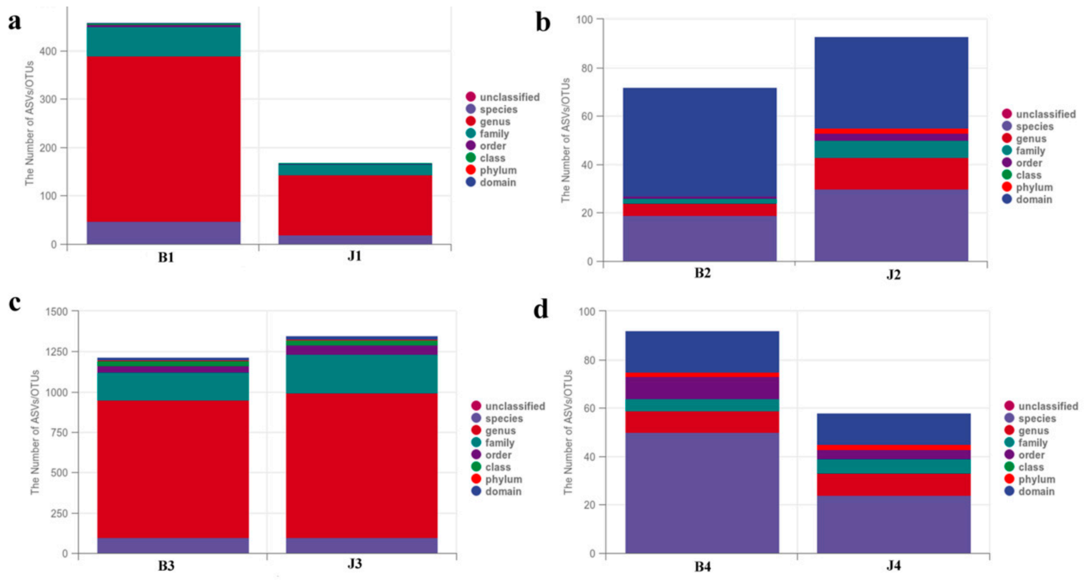

Figure S1. Species taxonomy annotated statistical chart.

Note: In the bacterial groups: tumor gummosis (B1) and healthy tissue (J1) at Bailuyuan; gummosis (B3) and healthy tissue (J3) at Zhouzhi. In the fungal groups: tumor gummosis (B2) and healthy tissue (J2) at Bailuyuan; gummosis (B4) and healthy tissue (J4) at Zhouzhi.

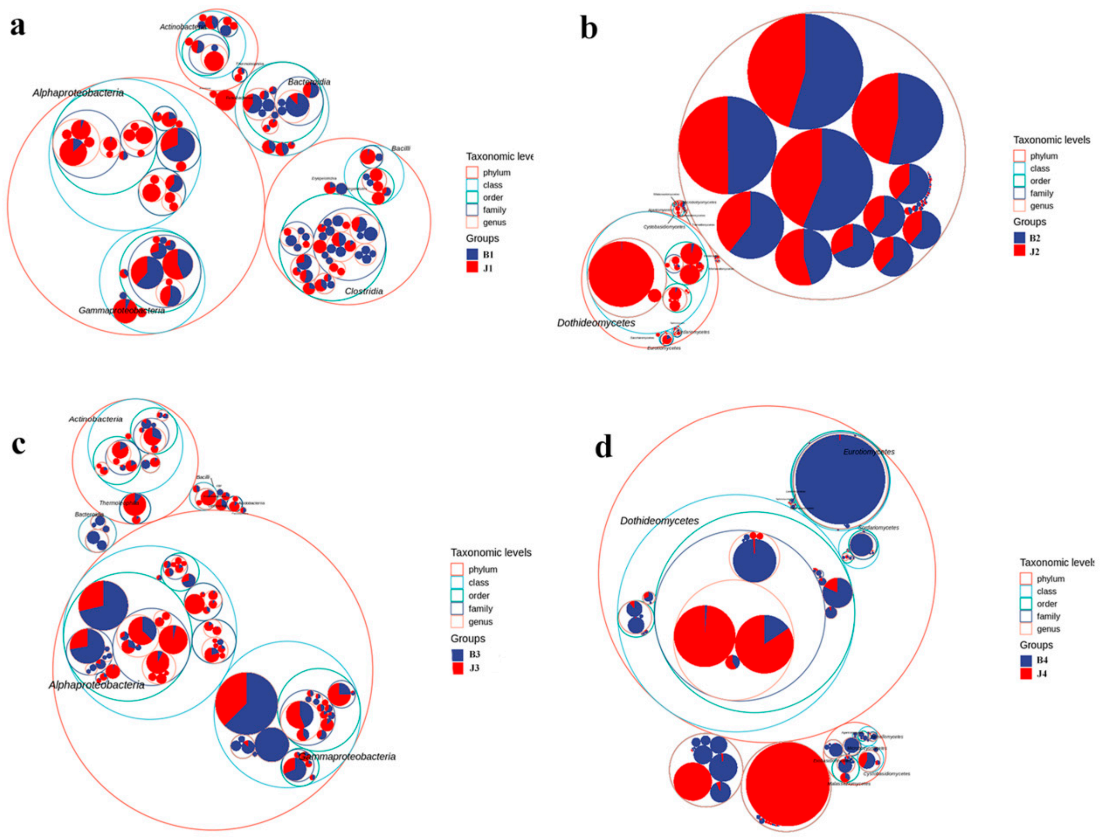

Figure S2. Classification level tree.

Note: In the bacterial groups: tumor gummosis (B1) and healthy tissue (J1) at Bailuyuan; gummosis (B3) and healthy tissue (J3) at Zhouzhi. In the fungal groups: tumor gummosis (B2) and healthy tissue (J2) at Bailuyuan; gummosis (B4) and healthy tissue (J4) at Zhouzhi.

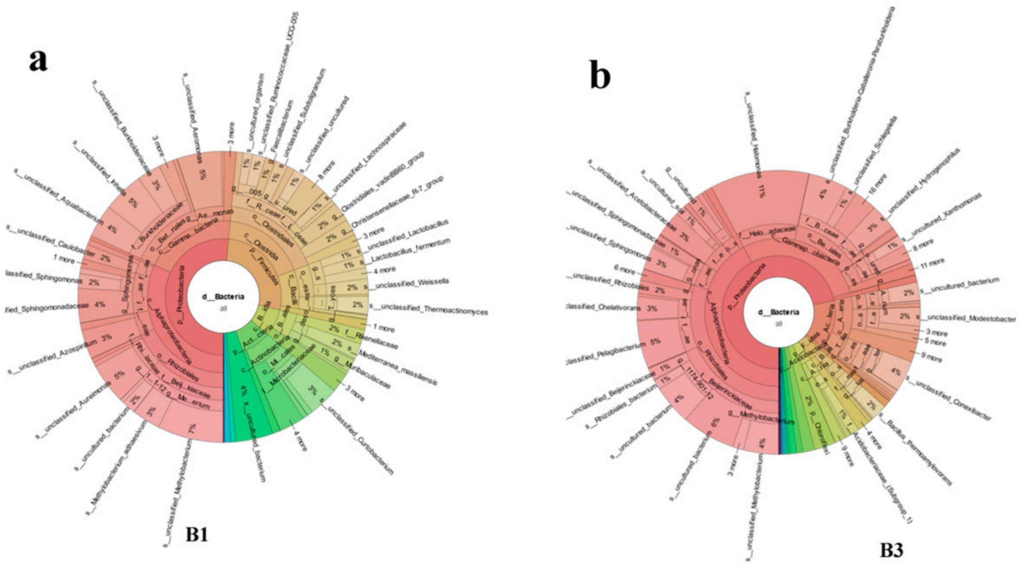

Figure S3. Krona classification of tissue bacteria during (a) tumor gummosis and (b) gummosis.

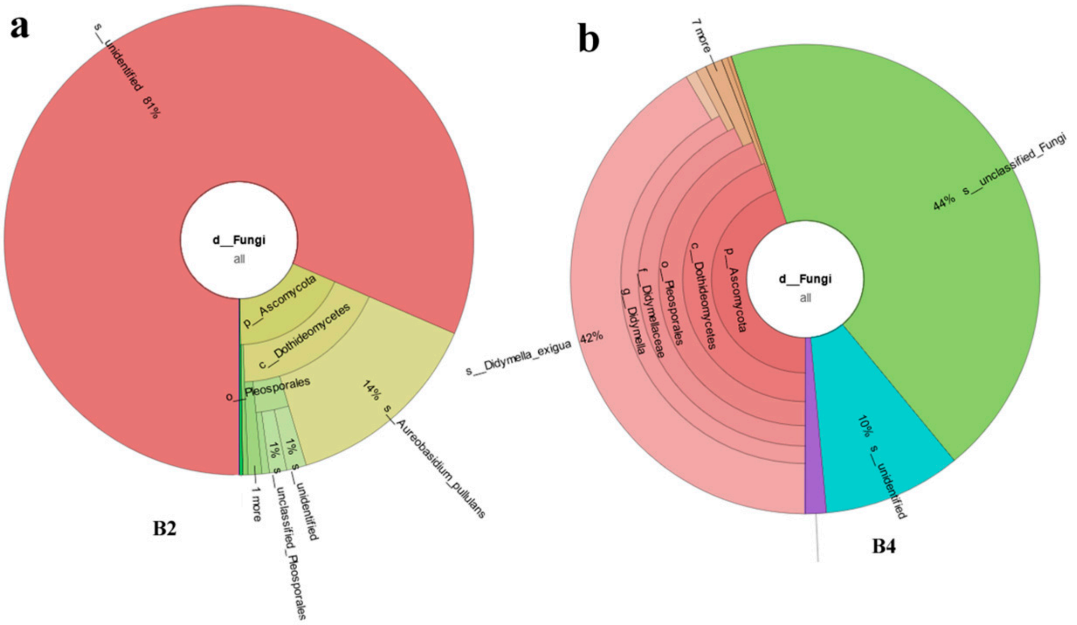

Figure S4. Krona classification of tissue fungi during (a) tumor gummosis and (b) gummosis.

Table S1. Statistics on sequencing volume and number of classification Units.

Sample	Input	Filtered	Denoised	Merged	Non-chimeric	Non-singleton	Sequence length	reads	ASVs	Domain	phylum	class	order	family	genus	species
J1	125809	116253	115390	114972	112296	112233	275-432	225939	502	1	7	12	28	47	69	16
B1	125664	117374	116136	115249	113643	113528	275-432			1	10	18	36	58	109	31
J2	144015	129920	129711	126704	114398	114395	161-247	223414	124	1	3	13	23	29	31	26
B2	146933	132228	131898	127707	109016	109012	161-247			1	4	12	20	23	22	19
J3	217115	204746	201314	196188	191811	191722	12-236	438850	2240	2	28	68	138	219	334	65
B3	270333	256113	253905	250795	247039	246982	129-236			2	27	68	138	218	346	85
J4	96188	87549	87464	87228	86090	86089	154-362	175511	116	1	2	11	16	26	26	21
B4	101065	92101	91973	91389	89421	89419	154-362			1	2	12	23	31	41	42
